# The Cost of Collection

**Published:** 1923-11

**Authors:** 


### THE COST OF COLLECTION.

In these days, when big organisations are employed to raise and co-ordinate revenue for the voluntary hospitals, a jealous eye is not unnaturally cast upon the expenses involved by their methods. We shall, indeed, probably require before long a uniform standard of accounts for such funds, as we do already for those of individual hospitals. Meantime, when the expenses of these funds are quoted, it is advisable to scrutinise carefully the items that the general term is made to cover.

### The Sheffield Report.

The first annual report of the Sheffield Joint Hospitals' Council, at which we glanced in our October issue, provides an instructive instance. Summarily considered, the accounts show a distribution of £52,000 to hospitals, and an expenditure of £10,000, making a collection of £62,000 in all. What does the £10,000 include? Half of it, or precisely £5,328, appears to be received by the Edgar Allen Institute in the form of a *per capitum* grant for the orthopædic treatment that the Institute provides for the contributors and their dependants, since its standing orders at present prevent the Institute from coming into the "pool." This then is "an expense," which happens to be half as much again as the sum paid by the Fund to the Sheffield Children's Hospital. But is not this "expense" as much a distribution to a hospital as those so classed in the accounts? Regarding it as such, the expenses are seen to be not £10,000 but £4,601, which also includes expenditure on equipment. This sum, moreover, is the initial expenditure incurred by starting the scheme, and ought to fall as the service and propaganda reap their natural fruits and the organisation gets a-going.

### A Low Ratio of Expense.

In sum, the percentage of expenses at present is 7½ of the income of the Association of Hospital Contributors. Even these expenses, however, include, beside the cost of collection (offices, salaries, printing and so forth), which are the strict "expense incurred" by the Fund, the cost of such services as union hospitals, ambulances and appliances, without which those who benefit by the Fund would be deprived of invaluable help. The general aim of all well-administered funds is to keep the rate of net expenditure upon "machinery" as much below 10 per cent. as possible. Generally speaking, 10 per cent. should be the limit for all funds, even in the early days of their creative endeavour. Other things being equal, and the cost of the different kinds of work they do allowed for, funds are economically administered in proportion to the degree to which they fall below this margin. We were careful last month to make no comment on the Sheffield Council's accounts, because such comment is misleading in the absence of definition and detail. These we glance at now, as the work of the Fund deserves, partly to confirm the confidence of its supporters, but even more to emphasise the need for a standard of account-keeping for such funds no less than for the separate hospitals.

